# Heat-Annealed Zinc Oxide on Flexible Carbon Nanotube Paper and Exposed to Gradient Light to Enhance Its Photoelectric Response

**DOI:** 10.3390/nano14090792

**Published:** 2024-05-02

**Authors:** Jih-Hsin Liu, Pi-Yu Shen

**Affiliations:** Department of Electrical Engineering, Tunghai University, Taichung 407, Taiwan; thusemidevice@gmail.com

**Keywords:** carbon nanotube paper, bucky paper, zinc oxide, rapid thermal annealing, photovoltaic response

## Abstract

Buckypaper (BP), a flexible and porous material, exhibits photovoltaic properties when exposed to light. In this study, we employed radio frequency (RF) sputtering of zinc oxide (ZnO) followed by rapid thermal annealing to enhance the photovoltaic response of BP. We investigated the impact of various sputtering parameters, such as the gas flow ratio of argon to oxygen and deposition time, on the morphology, composition, resistivity, and photovoltaic characteristics of ZnO-modified BP. Additionally, the photovoltaic performance of the samples under different illumination modes and wavelengths was compared. It was found that optimal sputtering conditions—argon to oxygen flow ratio of 1:2, deposition time of 20 min, and power of 100 watts—resulted in a ZnO film thickness of approximately 45 nanometers. After annealing at 400 °C for 10 min, the ZnO-modified BP demonstrated a significant increase in photocurrent and photovoltage, along with a reduction in resistivity, compared to unmodified BP. Moreover, under gradient illumination, the ZnO-modified BP exhibited a photovoltage enhancement of 14.70-fold and a photocurrent increase of 13.86-fold, compared to uniform illumination. Under blue light, it showed a higher photovoltaic response than under other colors. The enhancement in photovoltaic response is attributed to the formation of a Schottky junction between ZnO and BP, an increased carrier concentration gradient, and an expanded light absorption spectrum. Our results validate that ZnO sputtering followed by annealing is an effective method for modifying BP for photovoltaic applications such as solar cells and photodetectors.

## 1. Introduction

Carbon nanotubes (CNTs) are one-dimensional nanostructures distinguished by their exceptional electrical, mechanical, and optical properties. Since their discovery by Iijima in 1991 [1], considerable attention has been garnered by CNTs for their potential across a spectrum of applications, including sensors, transistors, solar cells, and flexible electronics. A significant challenge in harnessing the potential of CNTs lies in assembling them into macrostructures such as films, fibers, and papers, which retain their intrinsic properties and facilitate integration with other materials and devices. Among these structures, CNT papers, also known as buckypapers (BPs), consist of thinly veiled, randomly entangled CNTs and can be produced via the vacuum filtration of CNT suspensions [2,3,4,5]. In addition to other techniques, experiments have utilized the evaporation casting method and vacuum filtration technique to fabricate composites of carbon nanotubes and biopolymers. It was observed that altering the mass fraction of carbon nanotubes significantly influences the electrical and mechanical properties of the resulting buckypapers. This approach enables the tuning of the composite’s conductivity and mechanical strength, thereby accommodating diverse application requirements. In particular, the evaporation casting method is especially suited for preparing composites with high conductivity and a uniform distribution of carbon nanotubes [6,7]. BPs have been demonstrated to exhibit photovoltaic properties under various light sources and intensities, such as photocurrent generation and photoconductivity modulation [8,9]. Additionally, the high malleability of buckypaper renders it a promising candidate for flexible optoelectronic materials, as it can easily conform to various curved surfaces. This characteristic extends its applicability far beyond that of traditional rigid solid-state photodetectors, which are typically fabricated using thin film deposition techniques. However, the photovoltaic performance of BPs is constrained by their low light absorption and carrier concentration, which can be ameliorated by integrating additional materials like metal oxides that can act as light absorbers, charge carriers, or catalysts [10,11].

Zinc oxide (ZnO) is a widely utilized wide-bandgap semiconductor for optoelectronic applications such as dye-sensitized solar cells and photocatalysts [12,13]. ZnO offers several advantages, including high electron mobility, high transparency, low cost, and eco-friendliness [14,15,16,17]. ZnO can be deposited on various substrates, including CNTs, through different methods such as physical vapor deposition (PVD), chemical vapor deposition (CVD), and sputtering [18,19,20]. Sputtering is a versatile and controllable technique capable of producing uniform, high-quality ZnO films with adjustable properties like thickness, morphology, crystallinity, and doping [21,22]. It can also be employed to create gradient structures of ZnO on CNTs, inducing a gradient in carrier concentration and enhancing the composite’s photovoltaic response [23,24]. Furthermore, sputtering followed by subsequent thermal annealing can reduce defects, enhance crystallinity, and promote interfacial bonding, thereby improving the quality and performance of ZnO films [25,26].

In this study, we enhanced the photovoltaic characteristics of BPs by sputtering ZnO films onto them and subsequently annealing at various temperatures and durations. We investigated the impact of sputtering parameters, such as gas ratios, power, and time, on the morphology, composition, and resistivity of the ZnO films. Additionally, we measured the photocurrent and photovoltage of the ZnO/BP composites under different light sources, intensities, and modes, such as uniform and gradient illumination. We compared the photovoltaic performance of the ZnO/BP composites to that of the pristine BPs and discussed the potential mechanisms behind the observed phenomena. Our findings aim to optimize the ZnO/BP composite material for optoelectronic applications, showcasing the potential of BPs as flexible and functional substrates for nanomaterials.

## 2. Experimental Procedure

**Figure 1:**
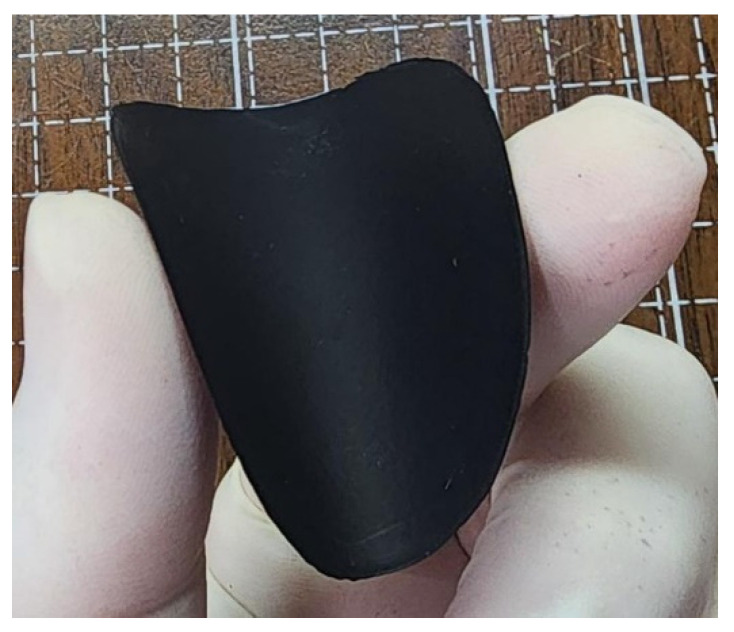
Photo of BP after the vacuum filtration process. BP is ductile, and MWCNTs are evenly distributed.

In this study, BP, consisting of approximately 0.06 g of multi-walled carbon nanotubes (MWCNTs), dispersed in a diluent containing the surfactant Triton X-100, was utilized. The MWCNTs were procured from CONJUTEK Corporation, model CRUDE, with a purity exceeding 95%, a specific surface area ranging from 150 to 250 m^2^/g, an average diameter of 10 to 20 nanometers, and lengths varying from 60 to 100 μm. Subsequently, ultrasonic disruption at a power of 50 watts was employed to disperse individual nanotubes, followed by a vacuum filtration process to complete the preparation procedure. The resulting BP had a diameter of approximately 4.5 cm, a thickness of about 75 μm, and a porosity of 78%, as depicted in Figure 1 below. Subsequently, the BP was sectioned into numerous rectangular pieces measuring 2.0 cm × 0.5 cm each for conducting experiments under various conditions.

The ZnO films were deposited on buckypaper (BP) using a pure zinc target (99.99%) with a diameter of 1.5 inches through the radio frequency (RF) sputtering (Junsun Tech. CS-400, New Taipei City, Taiwan) process. The vacuum chamber was initially evacuated to a pressure of 5 × 10^−5^ torr using a mechanical pump in conjunction with a turbo molecular pump, after which a mixture of argon and oxygen gases was introduced at different flow ratios. The working gas pressure during sputtering was maintained at 30 mtorr. The distance between the target and the substrate was set to 10 cm, and the substrate was mounted on an electric rotating platform spinning at 10 rotations per minute to ensure uniform deposition. The sputtering power was fixed at 100 watts, and the deposition times were varied at 5, 10, 20, and 40 min, respectively. To measure the thickness of the ZnO films, silicon substrates were placed alongside the BP substrates, and the thickness of the ZnO deposited on the silicon was measured using a profilometer, as shown in Table 1 below.

Later in our study, ZnO-modified BPs, which had been sputtered for 5, 20, and 40 min, underwent rapid thermal annealing (RTA) in a fast thermal processing system. The RTA vacuum quartz tube was evacuated to approximately 20 mtorr before the samples were rapidly heated using infrared lamps. The heating rate brought the temperature up to 400 °C in five minutes, where it was maintained for 10 min, after which the samples were cooled to room temperature via water circulation before being removed from the vacuum chamber.

The morphology and composition of the ZnO-modified BP were observed using a field emission scanning electron microscope (FESEM, JEOL JSM-7000F, Tokyo, Japan) and analyzed by energy dispersive X-ray spectroscopy (EDS, OXFORD X-ACT, Abingdon, UK). The crystalline structure of the ZnO-modified BP was determined through X-ray diffraction (XRD) analysis using a Philips X’Pert Pro MPD system with Cu Kα radiation. The resistivity of the ZnO-modified BP was measured using the four-point probe method with a Keithley 2400 (Cleveland, OH, USA) source meter. The photovoltaic characteristics of the ZnO-modified BP were assessed using a solar simulator (A.M. 1.5G Forter Tech. LCS-100, Taichung City, Taiwan) as the light source, with an intensity of 1000 W/m^2^. Photocurrent and photovoltage were recorded using a Keithley 2000 multimeter (Cleveland, OH, USA). The measurement errors associated with it are approximately 0.1 microvolts and 1 nanoampere, respectively. The samples were measured using two illumination modes: uniform illumination and gradient illumination. In the uniform mode, the entire area of the sample was exposed to the light source. For gradient illumination, the samples were exposed to a light source with a gradient of intensity, ranging from high to low across their area. This gradient illumination mode was achieved by placing gradient neutral density filters in front of the light source. Furthermore, the samples were measured under different wavelengths of light, ranging from 400 nm to 700 nm, utilizing red, green, and blue color filters to accomplish this.

## 3. Results and Discussion

**Figure 2:**
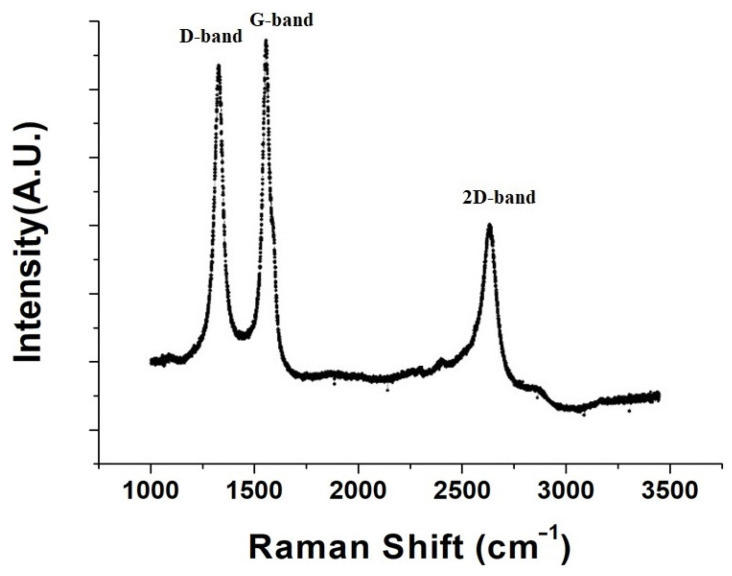
Raman spectroscopy graph of pure nanotube paper.

Figure 2 displays the Raman spectrum of pristine buckypaper (BP), showcasing two characteristic peaks of carbon nanotubes (CNTs): the D band at 1350 cm^−1^ and the G band at 1580 cm^−1^. The D band is associated with disorder and defects within the CNTs, while the G band relates to the in-plane vibrations of sp^2^ bonded carbon atoms. The intensity ratio of the D to G bands (ID/IG) reflects the crystallinity and purity of the CNTs. For pristine BP, the ID/IG ratio was found to be 0.99, indicating that the crystalline quality of the CNTs is reasonably acceptable.

**Figure 3:**
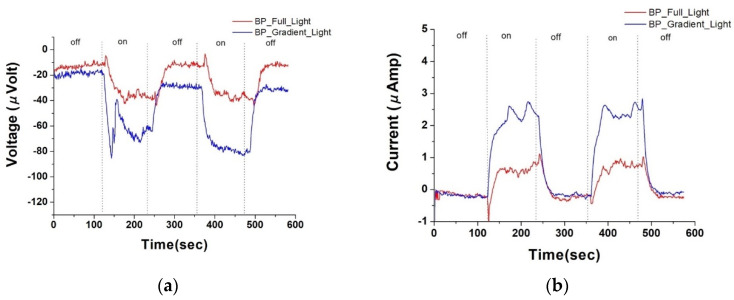
(**a**) Photovoltage response of pure buckypaper under full and gradient illumination; (**b**) photocurrent response of pure buckypaper under full and gradient illumination.

Figure 3a,b, respectively, depict the photovoltage and photocurrent of pristine buckypaper (BP) after a sputtering duration of five minutes, under both uniform and gradient illumination conditions. “On” in the figures refers to the state of illumination, while “off” indicates the absence of light. Due to the large surface area and internal structure of dangling bonds in BPs, self-charging phenomena are commonly observed during the measurement of its electrical properties. Additionally, as the light exposure cycles progress, the hysteresis effect intensifies, sometimes resulting in an initial voltage deviation when unexposed to light. This deviation is hypothesized to arise from charge redistribution and charge trapping effects within the material. In the case of BP, this is likely due to its rich surface chemical activity sites and complex microstructure, which facilitate the temporary capture of electrons at the material’s surface and interfaces. Such phenomena have been documented in the literature [8], particularly in carbon-based materials and other nanostructures with high surface activity. Therefore, when estimating photo-voltage or photo-current, this initial offset is used to recalibrate the measurements to zero. Therefore, under uniform illumination, pristine BP generated approximately −18.3 microvolts of photovoltage and 0.8 microamps of photocurrent; under gradient illumination, it produced approximately −35.20 microvolts of photovoltage and 2.60 microamps of photocurrent.

In Figure 3, at the onset of illumination, both the photovoltage and photocurrent display a very sharp peak. We hypothesize the possible mechanisms as follows:

Material and Junction Characteristics: These abrupt spikes, often described as transcendent phenomena in photoresponse, may be related to the specific properties of the materials used and the interaction at the junction between ZnO and buckypaper. In semiconductor devices, these spikes can originate from transient effects, where the rate of carrier generation temporarily exceeds the rate of recombination due to sudden changes in illumination. This leads to a transient excess of carriers, resulting in a surge in current or voltage.

Schottky Barrier Dynamics: The Schottky barrier formed at the interface between ZnO and buckypaper may also contribute to such peaks. The barrier influences the ease with which electrons can cross the junction. When illuminated, the barrier height or width might change abruptly, allowing a sudden flow of current before stabilization. This is consistent with described Schottky barrier mechanisms, where interactions of materials under varying light conditions can lead to changes in barrier properties, significantly affecting the current and voltage characteristics.

Thermionic Emission and Internal Photoemission: Depending on the materials and their work functions, these peaks may also result from thermionic emission or internal photoemission mechanisms. These processes are influenced by the photon energy striking the material and can, under specific conditions, cause a sudden increase in carrier mobility and density [27,28,29].

Equation (1), referencing the current density in semiconductor materials, demonstrates that the total current *J_p_* is the sum of the drift and diffusion currents. Upon illumination, numerous conductive electrons and holes are excited and subsequently moved by the internal electric field, generating the drift current *J_p,drift_*. Additionally, a concentration gradient causes an increase in the diffusion current *J_p,diffusion_* as reflected in the equation, thus enhancing the total current.

(1) $$J_{p}=J_{p,drift}+J_{p,diffusion}=qp\mu_{p}E-qD_{p}\frac{dp}{dx}$$

In this equation, *J_p,drift_* represents the drift current, *J_p,diffusion_* denotes the diffusion current, *q* is the elementary charge of the carrier, *p* indicates the concentration of the carriers, *μ_p_* is the mobility of the carriers, *E* represents the electric field applied across the semiconductor material, and *D_p_* is the diffusion coefficient for the carriers.

**Figure 4:**
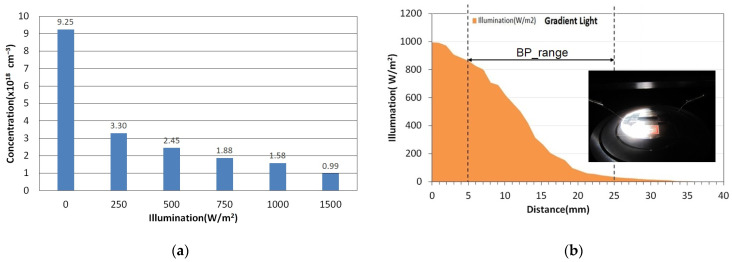
(**a**) Carrier concentrations measured in pure buckypaper under no illumination and under uniform illumination at intensities of 250, 500, 750, 1000, and 1500 W/m^2^. (**b**) The intensity of gradient illumination on buckypaper, varying with distance.

To verify that the carrier concentration in BP does indeed change under illumination, we manually adjusted the solar simulator to produce varying light intensities. Using the Hall effect method, we measured the carrier concentrations of pure BP at uniform light exposures of 0, 250, 500, 750, 1000, and 1500 W/m^2^, as illustrated in Figure 4a. Pure BP displayed a p-type semiconductor behavior, indicating that the predominant carriers were holes. As the light intensity increased, the carrier concentration decreased from 9.25 × 10^18^ cm^−3^ at zero illumination to 0.99 × 10^18^ cm^−3^ at 1500 W/m^2^. This decrease is hypothesized to result from the photogenerated electron–hole pair recombination, which reduces the number of free carriers in BP.

Furthermore, we introduced a gradient neutral density filter in front of the light source and slowly moved the light intensity sensor to monitor the changes in light intensity with distance, as shown in Figure 4b. This setup allowed us to further investigate the effects of varying light gradients on carrier behavior in BP under controlled conditions.

**Figure 5:**
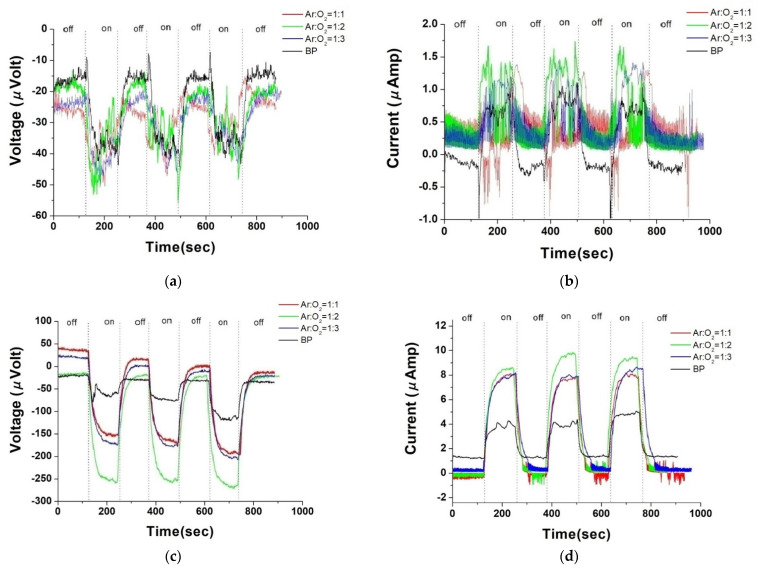
Photovoltage and photocurrent response under uniform illumination (**a**,**b**) and gradient illumination (**c**,**d**) of ZnO film deposited on buckypaper for 5 min at different argon and oxygen flow rate ratios.

Following the examination of the fundamental properties of pristine buckypaper (BP), we embarked on a series of experiments where ZnO material was deposited on BP using different ratios of argon to oxygen gas flow, at a power of 100 W, and for a sputtering duration of 5 min. We then examined the photovoltaic response of these ZnO/BP composites. Observing Figure 5a,b, a significant noise in the photovoltaic response is evident. This is postulated to stem from potential barriers at the material interface, formed when zinc oxide is deposited on buckypaper. Particularly under light intensities insufficient for all carriers to surmount these barriers, considerable voltage noise is generated. The research by Yoshida et al. discusses the 1/f noise in semiconductors, which originates from the random motion of carriers across potential barriers. As carriers move randomly between these barriers, fluctuations in local resistance occur, thus generating noise [30]. The use of gradient illumination conditions in the ZnO/BP composite material increases the internal concentration gradient differences, enhancing the diffusion current. This enhancement may help overcome the potential barriers introduced by the material interface, thereby reducing noise. Under gradient illumination conditions, Figure 5c,d exhibit smoother photovoltaic response curves. Figure 5a–d illustrate the comparison of photovoltage and photocurrent between pristine BP and ZnO/BP composites under both uniform and gradient illumination conditions. Under uniform illumination, due to grain boundary scattering by the ZnO material, both photocurrent and photovoltage exhibited more pronounced fluctuations during measurement. Conversely, under gradient illumination, the photovoltaic response curves were smoother, attributed to an enhancement in photovoltage energy. Furthermore, compared to pristine BP, the ZnO/BP composites showed a significantly improved photovoltaic response under gradient illumination. The peak values under various experimental conditions are summarized in the following bar graph.

**Figure 6:**
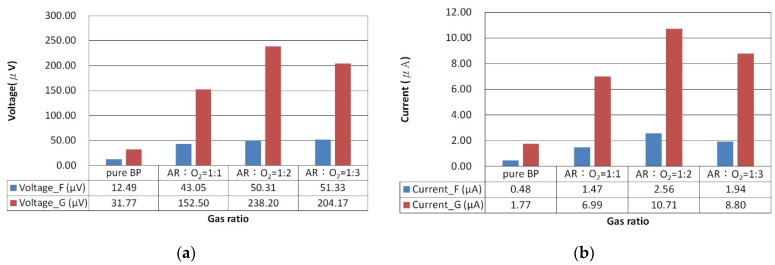
(**a**) Photovoltage and (**b**) photocurrent bar graph of ZnO film deposited for 5 min with a plasma power of 100 watts under different argon and oxygen flow rate ratios, under uniform and gradient illumination.

Figure 6 showcases the photovoltage and photocurrent of ZnO films deposited on BP substrates under different argon to oxygen gas flow ratios (ranging from 1:1 to 1:3), observed under both uniform and gradient illumination. For the ZnO/BP composites with an argon to oxygen gas flow ratio of 1:2, the highest photovoltage and photocurrent were recorded under gradient illumination, amounting to −238.20 microvolts and 10.71 microamps, respectively. In contrast, under uniform illumination, the values were only −50.30 microvolts and 1.13 microamps. Therefore, subsequent experiments involving thermal annealing of ZnO were conducted with the argon to oxygen gas flow ratio set at 1:2 for standard ZnO sputtering. The argon to oxygen gas flow ratio impacts the oxidation quality and carrier concentration of the ZnO films, as excess oxygen can decrease the sputtering rate and carrier density, while insufficient oxygen may result in suboptimal ZnO formation. The enhancement in photovoltaic response of the ZnO/BP composites is attributed to the ZnO material’s ability to extend the absorption spectrum, increase carrier concentration, and reduce impedance. The superior photovoltaic response under gradient illumination is due to an increase in diffusion current, resulting from higher concentration gradient differences within the ZnO/BP composites.

**Figure 7:**
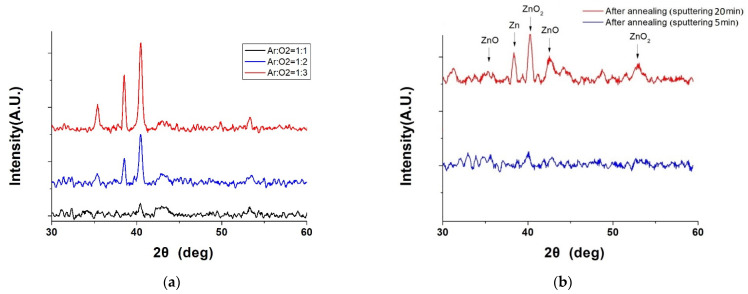
(**a**) XRD spectra of ZnO film deposited for 5 min under different argon and oxygen flow rate ratios and (**b**) XRD spectra of ZnO deposited under a flow rate ratio of argon to oxygen of 1:2 for 20 min, after annealing.

In addition to measuring the photovoltaic properties, we also sought to understand the crystalline phase changes in ZnO material during growth. Figure 7a displays the X-ray diffraction (XRD) patterns of ZnO films deposited on BP for 5 min at 100 W power under different argon to oxygen gas flow ratios. Due to the overwhelming peak intensities of the carbon material, which were not the focus of our study, their peaks are not shown. The XRD patterns reveal the crystalline structure and phase composition of the ZnO films. The XRD pattern for the argon to oxygen gas flow ratio of 1:3 showed optimal oxidation quality, as it exhibited the highest intensity peaks for ZnO at 35.3° and 42.8°, corresponding to the (002) and (101) planes of the hexagonal wurtzite structure of ZnO, respectively. The XRD pattern for a gas flow ratio of 1:1 displayed peaks for metallic zinc (Zn) at 38.5° and zinc peroxide (ZnO_2_) at 40.4° and 53.4°, indicating the incomplete oxidation of the zinc target. The pattern for a gas flow ratio of 1:2 showed a mix of ZnO, Zn, and ZnO2 peaks, suggesting medium oxidation quality.

The argon to oxygen gas flow ratio affects the oxidation of the zinc target, as excessive oxygen can decrease the sputtering rate, and insufficient oxygen can lead to suboptimal ZnO formation. However, since the photovoltaic characteristics of ZnO on BP at a gas flow ratio of 1:2 were superior to the other ratios, we continued to sputter ZnO material at this ratio for subsequent experiments.

Zinc oxide is a Group II–VI semiconductor, distinguished by its wide band gap of approximately 3.37 eV, which endows it with robust semiconducting properties at room temperature. This wide band gap also enables zinc oxide to exhibit favorable optical characteristics within the ultraviolet spectrum, while remaining largely unaffected by visible and infrared light. The crystal structure of zinc oxide is typically hexagonal, a configuration that facilitates effective electron and hole mobility, making it an excellent material for fabricating optoelectronic devices and sensors.

Zinc oxide possesses strong photoelectric properties, including photovoltaic effects and photoluminescence. Its ability to absorb ultraviolet light and generate a photocurrent makes it ideal for UV detectors. Additionally, zinc oxide demonstrates photoluminescence, particularly when doped or when structural defects are present, emitting blue or green light. Consequently, by modifying nanotube paper with zinc oxide, we have enhanced its photoelectric response, especially in terms of its reaction to blue light.

Figure 7b presents the XRD patterns of ZnO films before and after annealing at 400 °C, for a gas flow ratio of 1:2. The annealing process, applied to ZnO films deposited for 5 min, is presumed to reduce the intensity of ZnO peaks due to the lower mass of the material, not improving the crystallinity. Conversely, for ZnO deposited for 20 min, the post-annealing XRD pattern confirmed the presence of ZnO and CNTs in the ZnO/BP composite, as the ZnO peaks at 35.3° and 42.8° matched well with the CNT peaks at 26.0° and 43.9°, corresponding to the (002) and (100) planes of the graphite structure of CNTs. This indicates that the ZnO film infiltrated into the CNTs, forming a more uniform and compact composite material.

**Figure 8:**
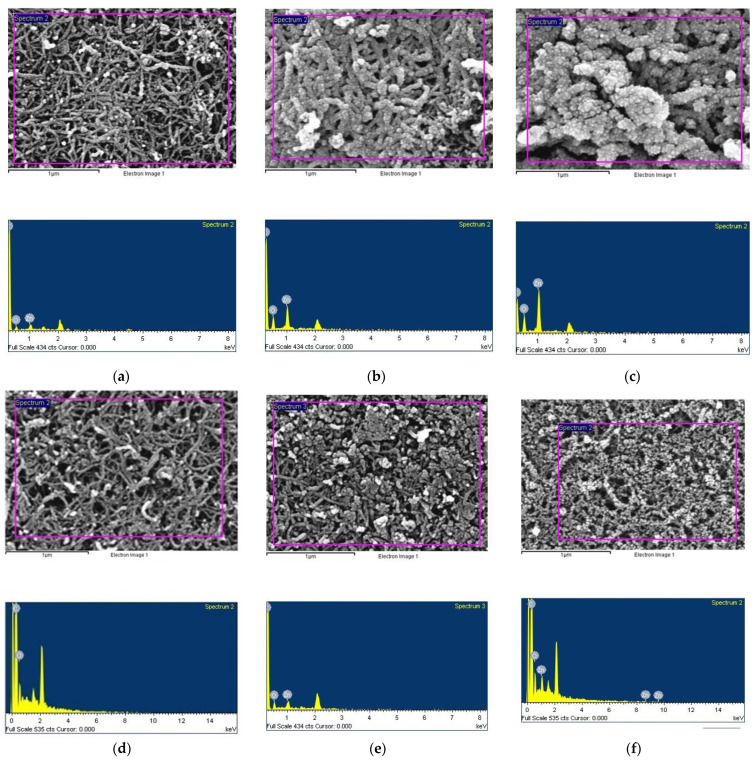
SEM images of ZnO sputtered on buckypaper at an argon and oxygen flow rate ratio of 1:2, (**a**–**c**), respectively, show films deposited for 5 min, 20 min, and 40 min without annealing, and (**d**–**f**) show films deposited for 5 min, 20 min, and 40 min with annealing.

To investigate the effects of annealing on ZnO films, we deposited ZnO on BP substrates for different sputtering durations (5 min, 20 min, and 40 min) with an argon to oxygen gas flow ratio of 1:2. Subsequently, the samples were annealed at 400 °C, and their surface morphologies were examined using scanning electron microscopy (FESEM, JEOL JSM-7000F, Tokyo, Japan), as shown in Figure 8a–f, while their elemental compositions and ratios were analyzed using energy dispersive X-ray spectroscopy (EDS), as presented in Table 2. SEM images reveal the morphology and thickness of the ZnO films, and EDS analysis confirms the elemental composition of the ZnO films.

With increasing sputtering time, the ZnO/BP surface sputtered for 5 min showed only a slight increase in crystalline grains. For the 20 min ZnO/BP, the carbon nanotubes were fully enveloped by ZnO grains, whereas for the 40 min ZnO/BP, the excessive ZnO coverage around the carbon nanotubes led to a significant increase in the average diameter of the nanotubes. The overly thick ZnO hindered light penetration to the carbon nanotubes, thereby reducing photovoltaic efficiency. Furthermore, after annealing, there was a noticeable change in the morphology of the ZnO films; they became thinner and more dispersed on the BP surface. EDS analysis indicated the presence of zinc and oxygen elements in the ZnO films. After annealing, the longer the ZnO sputtering time on BP, the greater the decrease in the ratio of oxygen to zinc on the surface. This reduction is attributed to the infiltration of ZnO into the CNTs and the partial evaporation and loss of material.

**Figure 9:**
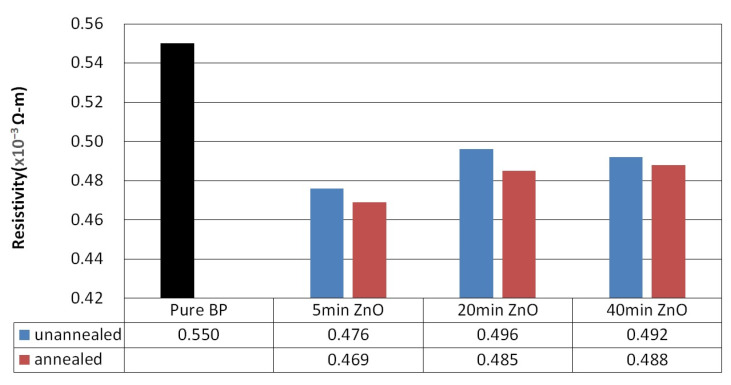
Resistivity of ZnO films deposited at different sputtering times with a plasma power of 100 watts, before and after thermal annealing.

Figure 9 illustrates the resistivity of ZnO films sputtered on BP substrates with an argon to oxygen gas flow ratio of 1:2, for varying sputtering durations (5 min, 20 min, and 40 min), both before and after annealing at 400 °C. Post annealing, the resistivity of the ZnO/BP composite materials decreased, from 4.76 × 10^−4^ ohm m for the 5 min sputtering duration to 4.69 × 10^−4^ ohm-m, from 4.96 × 10^−4^ ohm m for the 20 min duration to 4.85 × 10^−4^ ohm-m, and from 4.92 × 10^−4^ ohm m for the 40 min duration to slightly lower at 4.88 × 10^−4^ ohm-m. The reduction in resistivity after annealing is attributed to a denser structure and larger contact area of the ZnO/CNT composite material, which enhances the transport of carriers.

**Figure 10:**
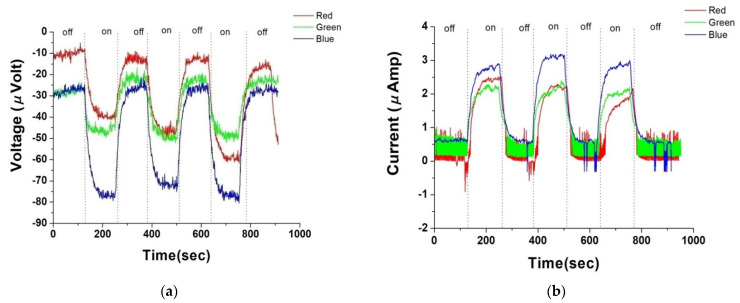
(**a**) Photovoltage and (**b**) photocurrent response under uniform illumination of different color lights on ZnO sputtered on buckypaper for 5 min with argon and oxygen at a 1:2 ratio.

Figure 10 presents the photovoltage and photocurrent of ZnO/BP composite materials with an argon to oxygen gas flow ratio of 1:2, under different wavelengths using RGB color filters. The ZnO/BP composites exhibited the highest photovoltage and photocurrent under blue light. The wavelength dependency of the photovoltaic response of the ZnO/BP composites is attributed to the energy level alignment of the ZnO/CNT heterostructure, which aligns well with blue and ultraviolet light.

**Figure 11:**
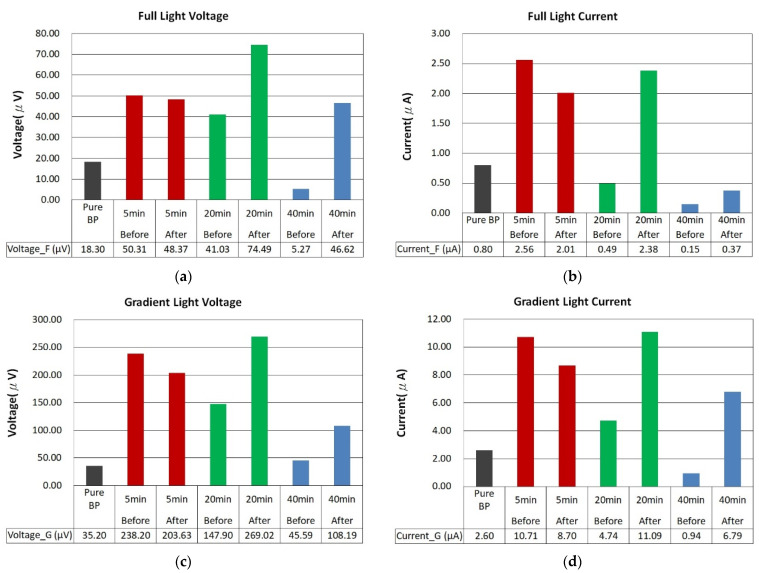
Comparison of photovoltage and photocurrent before (**a**,**b**) and after (**c**,**d**) annealing of ZnO films deposited on buckypaper at an argon and oxygen flow rate ratio of 1:2 for 5 min, 10 min, and 40 min.

The photovoltaic performance after thermal annealing, as shown in Figure 11a–d, depicts the photovoltage and photocurrent for ZnO films sputtered on BP substrates with an argon to oxygen gas flow ratio of 1:2, across different sputtering durations (5 min, 20 min, and 40 min), before and after annealing at 400 °C, under both uniform and gradient illumination. Before annealing, the ZnO/BP composites with a 5 min deposition time showed commendable photovoltage and photocurrent, attributed to the infiltration of the ZnO film into the CNTs and the elimination of some impurities. This improved the carrier contact and transport within the ZnO/CNT composite material. However, after annealing, a slight decrease was observed. Based on SEM images from Figure 8a,d, it is speculated that the reduced coverage and quantity of ZnO, combined with material loss during annealing, resulted in the remaining scant ZnO failing to further enhance the photovoltaic effect.

Upon closer inspection of the samples with a 20 min ZnO deposition time, the photovoltage increased from 18.30 microvolts to 41.03 microvolts and then rose to 74.49 microvolts after annealing. Subsequent exposure to gradient illumination further enhanced the photovoltaic effect, ultimately increasing the photovoltage by a total of 14.70-fold to 269.02 microvolts. Similarly, the photocurrent saw an overall increase of 13.86-fold, reaching 11.09 microamps. In contrast, the samples with a 40 min ZnO deposition time did not exhibit an enhanced photovoltaic effect due to the excessive modifier material blocking light from reaching the internal BP/ZnO junctions.

From Figure 11a,c, the photovoltage of the unmodified buckypaper was approximately 18.30 microvolts. After the application of zinc oxide through sputtering, there was a modest increase in photovoltage to 41.03 microvolts. Subsequent annealing further enhanced the photovoltage to 74.49 microvolts. Exposure to a gradient of light intensities dramatically raised the photovoltage to 269.02 microvolts. By subtracting the photovoltage values at each stage of the experiment, we can also discern the incremental contribution of each experimental condition to the enhanced photovoltage response.

For ZnO/BP composite materials, the optimal sputtering duration was found to be 20 min, as it showed the highest photovoltage and photocurrent after annealing under both lighting modes. The increase in photovoltage and photocurrent can be attributed to the thicker ZnO film and the formation of the ZnO/CNT heterostructure, enhancing light absorption and carrier separation. The slight decrease in photovoltaic response is due to the reduced light transmittance and increased recombination rate associated with overly thick ZnO films. The higher photovoltaic response of ZnO/BP composites under gradient illumination compared to uniform illumination is due to the increased diffusion current, resulting from higher concentration gradient differences within the ZnO/BP composite material.

## 4. Conclusions

In this study, we fabricated flexible ZnO/BP composite materials by sputtering ZnO onto BP under optimized parameters. The optimal sputtering conditions were identified as a gas flow ratio of argon to oxygen at 1:2, with a duration of 20 min, followed by an annealing process at 400 °C for 10 min. This modification of BP by ZnO extended its absorption spectrum under blue light, increased the carrier concentration, and reduced its electrical resistance. Furthermore, under gradient illumination, this composite exhibited a higher photoresponse compared to uniform illumination.

Although the ZnO/BP composite materials possess the attributes of flexible electronic materials, thus broadening their application spectrum beyond that of rigid solid-state photodetectors, their increased surface area and multiple dangling bonds contribute to a slight self-charging effect. It is conceivable that enhancing the conductivity during the modification process could accelerate the charge–discharge recovery time, thereby perfecting the characteristics of flexible photoelectric materials. We believe that the ductility, lightweight nature, and enhanced photoelectric performance of the ZnO/BP composites indicate them to be candidates with potential and versatile applications in solar cells and photodetection devices.

## Figures and Tables

**Table 1:** Film thickness of ZnO deposited at different sputtering times.

ZnO Film Thickness				
Deposition time (min)	5	10	20	40
Film thickness(nm)	10.21	19.83	44.52	80.25

**Table 2:** Weight percentage of elements of EDS analysis of films sputtered with ZnO on buckypaper at an argon and oxygen flow rate ratio of 1:2, deposited for 5 min, 20 min, and 40 min, before and after annealing.

EDS	Sputtering 5 min		Sputtering 20 min		Sputtering 40 min	
	Before Annealing	After Annealing	Before Annealing	After Annealing	Before Annealing	After Annealing
Oxygen	5.73%	5.48%	13.59%	7.57%	22.05%	9.64%
Zinc	1.51%	1.08%	4.13%	1.83%	11.83%	3.88%

## Data Availability

Data are contained within the article.
